# Metagenomic next-generation sequencing-guided antimicrobial treatment versus conventional antimicrobial treatment in early severe community-acquired pneumonia among immunocompromised patients (MATESHIP): A study protocol

**DOI:** 10.3389/fmicb.2022.927842

**Published:** 2022-08-02

**Authors:** Shaohua Fan, Min Si, Nana Xu, Meichen Yan, Mingmin Pang, Guangfeng Liu, Jibin Gong, Hao Wang

**Affiliations:** ^1^Department of Critical Care Medicine, Central Hospital Affiliated to Shandong First Medical University, Jinan, China; ^2^Department of Cardiac Surgery, Cardiac Surgery Care Unit, Qilu Hospital of Shandong University, Jinan, China; ^3^Department of Critical Care Medicine, Qilu Hospital of Shandong University, Jinan, China

**Keywords:** metagenomic next generation sequencing, immunosuppression, immunocompromised patient, community-acquired pneumonia, antimicrobial therapy

## Abstract

**Background:**

Severe community-acquired pneumonia (SCAP) is the main cause of mortality in immunocompromised patients. Compared with conventional microbiological tests (CMT), metagenomic next-generation sequencing (mNGS) can quickly and simultaneously detect a wide array of bacteria, viruses, and fungi in an unbiased manner. It is increasingly used for severe respiratory infectious diseases, especially for immunocompromised patients. However, the effects of mNGS-based antimicrobial treatment procedures on clinical outcomes in immunocompromised patients with SCAP have not been evaluated.

**Methods/Design:**

The MATESHIP study is a prospective, multicenter, parallel-group, open-label, randomized controlled trial from 20 ICUs in university hospitals and academic teaching hospitals across Shandong Province, China. We will enroll 342 immunocompromised patients with early onset SCAP who are admitted to an intensive care unit (ICU). Participants will be randomly allocated to an mNGS-guided treatment group or a conventional treatment group (guided by CMT), according to centrally computer-based block randomization stratified by participating centers. Participants will undergo CMT tests using appropriate lower respiratory tract (LRT) and other necessary specimens, with or without mNGS tests using LRT specimens. The primary outcomes will be: (1) The relative change in Sequential Organ Failure Assessment (SOFA) score from randomization to day 5, day 7, day 10, or the day of ICU discharge/death; and (2) the consumption of antimicrobial agents during ICU stay (expressed as defined daily doses). The secondary outcome measures will be: days from randomization to initiation of definitive antimicrobial treatment; overall antimicrobial agent use and cost; total cost of hospitalization; length of ICU stay; 28- and 90-day mortality; and clinical cure rate. This study hypothesizes that mNGS-guided treatment will decrease the degree of organ dysfunction/failure, the consumption of antimicrobial agents, and mortality, while the cure rate will be increased, and the time to initiation of appropriate therapy will be advanced.

**Discussion:**

The MATESHIP study will evaluate for the first time whether mNGS-guided antimicrobial therapy improves the outcomes of SCAP in an immunocompromised population, and provide high-level evidence on the application of mNGS in the management of this population.

**Clinical Trial Registration:**

[ClinicalTrials.gov], identifier [NCT05290454].

## Introduction

Severe community-acquired pneumonia (SCAP) is the primary cause of mortality in immunocompromised patients (Jain et al., 2015; Metlay et al., 2019; Azoulay et al., 2020; Aliberti et al., 2021). It is also a leading cause of intensive care unit (ICU) admission (approximately 20–30%) (Ramirez et al., 2020). There is a global increase in patients with distinct immunocompromising conditions, because of the advance of cancer treatment, increasing development of immunosuppressants for autoimmune diseases, and organ transplants (Cheng et al., 2021; Fung and Babik, 2021).

The mortality rate for immunocompromised patients with SCAP can be up to 50% (Aliberti et al., 2021); the disease course can be complicated, including respiratory failure and sepsis. The major reasons for this high mortality are the high risk from mixed and unusual pathogens, and delayed initiation of appropriate antimicrobial therapy (Garnacho-Montero et al., 2018; Nair and Niederman, 2020; Aliberti et al., 2021; Sun et al., 2021; Zhao et al., 2021). Timely identification of the causative microorganism(s) is crucial for early initiation of antimicrobial treatment, which is the most effective measure to improve the clinical prognosis and reduce the mortality of immunocompromised patients with SCAP (Metlay et al., 2019; Azoulay et al., 2020; Ramirez et al., 2020).

Conventional microbiological tests (CMT) such as standard cultures, microscopy, PCR, pathogen-specific antigen tests, and antibody assays, are commonly used to detect pathogens. However, they have various limitations, such as long turnaround times, low detection rates, and inability to be used for certain pathogens (Jain et al., 2015; Azoulay et al., 2020; Aliberti et al., 2021; Azar et al., 2021). Metagenomic next-generation sequencing (mNGS) can quickly (usually within 24 h) and simultaneously detect a wide array of bacteria, viruses, and fungi in appropriate lower respiratory tract (LRT) specimens in an unbiased manner by analyzing nucleic acid fragments of the pathogens (Chiu and Miller, 2019). This methodology is increasingly being used for severe respiratory infectious diseases, especially among immunocompromised patients (Parize et al., 2017; Langelier et al., 2018; Pan et al., 2018; Gu et al., 2019; Azar et al., 2021; Zhan et al., 2021). Recent clinical studies from us and other researchers have confirmed the value of mNGS, compared with CMT, in detecting pathogens, including a shorter detection cycle, higher sensitivity and specificity, and greater ability to detect rare or special pathogens (Parize et al., 2017; Zinter et al., 2019; Li et al., 2020; Yin et al., 2022). Moreover, the use of mNGS enables better targeted use of antimicrobial agents in immunocompromised patients. In a prospective observational study of 75 immunocompromised patients, mNGS results had an impact on treatment in 52% of the patients (de-escalation or initiation of targeted treatment) (Zhan et al., 2021). In another retrospective single-center study, more than half (13 of 23) of SCAP patients in the immunosuppressed group had reduced or downgraded antibiotic treatment based on the results (Sun et al., 2021).

However, little is known about the effects of mNGS-based antimicrobial adjustment on clinical outcomes in immunocompromised patients with SCAP (Garnacho-Montero et al., 2018; Torres et al., 2019). High-quality evidence is needed to determine the clinical benefits of mNGS-guided antimicrobial therapy compared with those of conventional antimicrobial treatment. We therefore designed this prospective, multicenter, randomized controlled trial; 342 immunocompromised patients with SCAP will be enrolled. We aim to explore the effect of mNGS-guided antimicrobial treatment versus conventional treatment in early SCAP among immunocompromised patients (MATESHIP study, *n* = 171 per arm of the trial). It is postulated that the degree of organ dysfunction/failure, the consumption of antimicrobial agents, and mortality will be decreased, the cure rate will be increased, and the time to initiation of appropriate therapy will be advanced in the mNGS-guided antimicrobial treatment group.

## Materials and methods

### Study design and setting

The MATESHIP study is designed as a prospective, multicenter, parallel-group, open, randomized controlled trial that will recruit a maximum of 342 immunocompromised participants with SCAP from 20 ICUs in university hospitals and academic teaching hospitals across Shandong Province, China, and is expected to last for 1 year. The study was reviewed and approved by the Medical Ethics Committee of Qilu Hospital of Shandong University (approval no. KYLL-202204-020), and was registered on ClinicalTrials. gov (registration No. NCT05290454). The enrollment, intervention, and assessment processes are shown in Supplementary Figures 1, 2.

### Participants

We will include immunocompromised patients with early SCAP. Participants will be enrolled if they meet the following inclusion criteria: (1) Diagnosed with SCAP and admitted to an ICU; (2) time from SCAP diagnosis to ICU admission < 24 h; and (3) the patient is immunocompromised. Informed consent will be obtained from all the patients or their guardians before enrollment. Participants will be excluded if they are: (1) Aged < 18 years; (2) pregnant or lactating; (3) are expected to die within 72 h; or (4) are receiving palliative therapy or supportive treatment only.

### Definitions

Severe community-acquired pneumonia is defined as present in patients with either one major criterion or at least three minor criteria according to the 2019 American Thoracic Society (ATS)/Infectious Diseases Society of America (IDSA) community-acquired pneumonia (CAP) severity criteria (Metlay et al., 2019): Major criteria: (1) Septic shock with need for vasopressors; (2) respiratory failure requiring mechanical ventilation. Minor criteria: (1) Respiratory rate > 30 breaths/min; (2) PaO_2_/FIO_2_ ratio < 250; (3) multilobar infiltrates; (4) confusion/disorientation; (5) uremia (blood urea nitrogen level > 20 mg/dl); (6) leukopenia (white blood cell count < 4,000 cells/μl); (7) thrombocytopenia (platelet count < 100,000/μl); (8) hypothermia (core temperature < 36°C); (9) hypotension requiring aggressive fluid resuscitation.

According to previous studies (Azoulay et al., 2018, 2020), immunocompromised conditions are defined as: (1) Use of long-term (>3 months) or high-dose (>0.5 mg/kg/d) steroids; (2) use of other immunosuppressant drugs; (3) solid organ transplantation; (4) solid tumor requiring chemotherapy in the last 5 years; (5) hematologic malignancy regardless of time since diagnosis and received treatments; (6) primary immune deficiency; (7) HIV infection with a CD4 T-lymphocyte count < 200 cells/ml or < 14%; (8) laboratory tests showing absolute neutrophil count < 1.000 × 10^9^/l on ICU admission; or (9) other immunosuppressed status judged by the physicians.

### Randomization

The randomization will be done centrally by a computer-generated sequence, with a stratification based on the center, and varying block sizes of four, six, and eight. Participants will be allocated in a ratio of 1:1 to the intervention group or control group according to the computer-generated list produced by the central center. Local physicians in each center will be responsible for inclusion of participants. The investigator in each center will contact the central center and participants will be allocated within 6 h of admission to the ICU. The day of randomization is defined as day 0.

### Blinding

This study is designed to compare the effects of two non-pharmacological treatment procedures on immunocompromised patients with SCAP. Considering the nature of the intervention, this study is open to the clinicians, patients, and investigator; only the data analyst and follow-up evaluator will be blinded.

### Study interventions

Participants in the MATESHIP study will be randomly allocated to receive mNGS-guided treatment based on both results of mNGS and CMT (the experimental arm); or conventional treatment only based on the results of CMT (the control arm).

### Control arm: Conventional treatment group

In the conventional treatment group, clinicians will alter or confirm definitive treatment based on the results of CMT. Participants will undergo CMT using appropriate LRT specimens and other necessary specimens (such as blood, pleural fluid, urine, et al.). LRT specimens including endotracheal aspiration, bronchoalveolar lavage fluid (BALF), and protected specimen brush, will be obtained within 24 h of participants entering the ICU. Blood samples, mid-stream urine, pleural fluid, and other respiratory specimens will be collected as soon as possible after admission and, preferably, before antimicrobial therapy begins. CMT, including bacterial/fungal stains and cultures, single or multiple RT-PCR, blood culture, serum and urine pathogen-specific antigen tests, and serum pathogen-specific antibody tests, will be performed according to the consensus statement regarding the management of immunocompromised patients with CAP and participant conditions.

### Experimental arm: Metagenomic next-generation sequencing-guided treatment group

In the mNGS-guided treatment group, clinicians will alter or confirm definitive treatment based on both mNGS and CMT results. Participants will undergo mNGS tests using appropriate LRT specimens. CMT will also be carried out using appropriate LRT specimens and other necessary specimens (such as blood, pleural fluid, urine, et al.), as described for the control group. LRT specimens will be divided into aliquots and used for both mNGS tests and CMT.

### Patient management

As immunocompromised patients have different types of pre-existing immune dysfunction and unique immunological risk, they often need individualized empirical treatments. According to a consensus statement regarding initial strategies for immunocompromised patients with CAP (Ramirez et al., 2020) and 2021 International Guidelines for Management of Sepsis and Septic Shock (Evans et al., 2021), the participants will receive individualized therapy depending on the clinical status, medical history, laboratory results and imaging patterns (details shown in Supplementary Materials). When we get the results of microbiologist tests, we will alter or confirm a definitive treatment. If the participants complicate with sepsis or septic shock, they will be managed according to the 2021 International Guidelines (Evans et al., 2021). All the participants will receive the best standard of care, according to the usual practice of the local intensivists.

### Microbiological tests

Conventional routine microbiological tests will be performed in local laboratories. LRT samples for mNGS tests will be transferred to the same professional genomic laboratory independently by cold-chain transportation; the genomic laboratory will perform nucleic acid extraction, library construction, amplification and sequencing, bioinformatic analysis, and data interpretation according to previous clinical practice (Li and Durbin, 2009; Miao et al., 2018; Gu et al., 2019). During our study period, the professional genomic laboratory will use the consistent mNGS detection protocol among the samples of enrolled patients. The results of mNGS will be considered positive when they meet criteria according to previous studies (Schlaberg et al., 2017; Li et al., 2018; Miller et al., 2019; Peng et al., 2021). Bacteria (mycobacteria excluded) and fungi will be considered pathogens (species level) when the reads per million (RPM) ratio, or RPM-r was ≥ 5, or > 30% relative abundance at genus level. Virus is considered positive as the coverage of three or more non-overlapping regions on the genome. Mycobacterium tuberculosis (MTB) is considered positive when at least 1 read was mapped to either the species or genus level.

An independent multidisciplinary panel of senior experts, including one infectious disease specialist, an intensivist, and a microbiologist, independently adjudicate the causative microorganisms for each patient after reviewing the mNGS results and necessary clinical data.

### Data collection and outcome measures

Trained research investigators will collect data and record it on the case report form (CRF). Baseline characteristics, Pneumonia Severity Index (PSI) score, CURB-65 (C: disturbance of consciousness, U: urea nitrogen, R: respiratory rate, B: blood pressure, 65: age) score, Acute Physiology and Chronic Health Evaluation II (APACEII) score, and Sequential Organ Failure Assessment (SOFA) score will be collected at the time of ICU admission. Laboratory test results, imaging findings, and management, including antimicrobial therapy and organ support, will be collected during hospitalization. The SOFA score is used to describe quantitatively and as objectively as possible the degree of organ dysfunction/failure over time. We will record the worst value of the SOFA score from randomization until day 10 or the day of ICU discharge/death. The score value is 0–24, and a higher score value indicates a worse outcome.

The primary outcomes of this study will be: (1) The relative change in SOFA score from randomization to day 5, day 7, day 10, or the day of ICU discharge/death; and (2) the consumption of antimicrobial agents during ICU stay (expressed as defined daily doses). The secondary outcome measures will be: days from randomization to initiation of definitive antimicrobial treatment; overall antimicrobial agent use and cost; total cost of hospitalization; length of ICU stay; 28- and 90-day mortality; and clinical cure rate. The outcome measures will be collected at the end of study to evaluate clinical and economic efficacy.

Data will be entered into SPSS software by trained staff. We will set up parameters to minimize the chance of data entry errors, and missing data or suspected errors should be resolved before data analysis. Investigators and data entry staff will be trained by Qilu Hospital Clinical Studies Center.

### Sample size calculation

According to a previous report (Laterre et al., 2019), we assumed that the mean relative change in SOFA score from randomization to day 5 in the conventional treatment group would be 12.1% with a standard deviation of 31.2%. The relative change in SOFA score from randomization to day 5 in the mNGS-guided treatment group is assumed to be 22.1%. The appropriate sample size was thus calculated, using a two-sided *Z*-test, a significance level of 0.0500, and a power of 80%. We plan to include a total of 318 participants (154 participants in each arm of the trial). To allow for an estimated 10% attrition, the total number of included participants will be 342. To recruit sufficient participants, this study will be conducted in 20 ICUs of university hospitals and academic teaching hospitals across Shandong Province.

### Statistical methods

All statistical analyses will be performed using SAS and R^1^ software. All statistical tests will use two-sided tests and a *P*-value < 0.05 will be considered statistically significant, unless otherwise specified.

Baseline characteristics will be reported. Comparisons of continuous variables between two groups will be performed using Student’s *t*-test. Categorical variables will be compared by using chi-square tests. Multivariate analysis (the Cox proportional hazards regression model) will be used to estimate hazard ratios adjusted for age, severity of illness, and underlying disease category. The primary outcomes between the two groups will be tested based on the intention-to-treat principle using a *t*-test. The secondary outcome measures will be compared between two groups as time-to-event variables using a log-rank test.

### Missing value analysis

Main outcome measurements are not expected to be missing values. Participants who withdraw from the study or are lost to follow up will be excluded from the study and included in the dropped cases. The total dropout rate between groups and the dropout rate due to adverse events will be compared using the chi-square test.

### Data monitoring committee

A data monitoring committee (DMC), comprising one infectious disease specialist, an intensivist, a microbiologist, and a statistician, will be established. The DMC will review data on patient characteristics, compliance, and study outcomes on a half-year basis, and make recommendations regarding continuation, modification, or discontinuation of the clinical trial.

The safety of the study will be assessed through reporting of adverse events, serious adverse events (SAEs), and suspected unexpected serious adverse reactions (SUSARs). The DMC will receive the analysis reports of adverse events to assess the safety of the intervention. However, this is a study of non-pharmacological treatment procedures—in theory, there will be no SAEs and SUSARs. The only likely adverse events are side effects of drugs used during treatment.

## Results

The MATESHIP study hypothesizes that mNGS-guided treatment will decrease the degree of organ dysfunction/failure, the consumption of antimicrobial agents, and mortality, while the cure rate will be increased, and the time to initiation of appropriate therapy will be advanced. This study will provide high-level evidence on the application of mNGS and the management of this special SCAP population.

## Discussion

Immunocompromised patients are vulnerable to specific pathogens, multidrug-resistant organisms, and multiple-microorganism infections (Di Pasquale et al., 2019). We are not aware of the common microbiology of immunocompromised patients with SCAP. Hence, the most common management strategies may not be suitable for these patients (Aliberti et al., 2021). Neither the 2019 ATS/IDSA guidelines nor the 2016 Clinical Practice Guidelines of the Chinese Thoracic Society recommend how to manage immunocompromised patients with SCAP, and recommendations for routine antimicrobial treatment in the initial stage are scarce (Cao et al., 2018). The initial antibiotic therapy may be a clinical failure if it excludes common microbes or there are multidrug-resistant organisms present (Di Pasquale et al., 2019; Ramirez et al., 2020).

Conventional microbiological tests have with various limitations, for example, standard cultures have long turnaround times and low positivity rates, and they may be unable to detect fastidious organisms. Molecular assays and antigen tests are limited to the detection of individual or several pathogens. Antibody-based tests may be insensitive in immunosuppressed patients because of poor antibody responses. A large, prospective study of CAP in hospitalized adults in the United States showed that the causative agent was not detected using CMT in 62% of participants (Jain et al., 2015). The difficulties in the detection/identification of pathogens pose a huge challenge for the management of SCAP, especially in immunocompromised patients (Sousa et al., 2013; Azoulay et al., 2019).

Immunocompromised patients have a more complex microbial etiology and are susceptible to uncommon pathogens or coexisting infections (Di Pasquale et al., 2019; Peng et al., 2021; Zhan et al., 2021). mNGS has advantages in detecting pathogens because of its high-throughput capacity, fast turnaround time (usually within 24 h) and ability to simultaneously detect a wide array of bacteria, viruses, and fungi in an unbiased way (Diao et al., 2022). Among the patients with community-acquired pneumonia or undefined pneumonia, mNGS using lower respiratory tract samples has a distinct advantage in the field of fungi or viral identification (such as *Pneumocystis jirovecii*, cytomegalovirus, et al.) and co-infection (such as *Acinetobacter baumannii* co-infected with *pneumocystis jirovecii* or cytomegalovirus, *Aspergillus* co-infected with cytomegalovirus), when comparing with the conventional microbiologist testing (CMT) (Pan et al., 2018; Sun et al., 2021). Other recent clinical studies also disclosed that mNGS showed remarkable advantages in terms of uncommon opportunistic pathogens (such as *Mucormycosis*, *Nocardia*) (Li et al., 2018; Azar et al., 2021; Zhan et al., 2021). In patients with lower respiratory tract infections (LRTI) in hematopoietic cellular transplant (HCT) recipients (Langelier et al., 2018), pathogens identified by CMT were all detected by mNGS, in contrast, mNGS identified well recognized respiratory pathogens, including human coronavirus 229E and human rhinovirus A, which are leading causes of CAP (Jain et al., 2015), but were negative by CMT and are still not listed in many clinical respiratory viral PCR panels. Moreover, mNGS identified several potential bacterial pathogens, such as *Streptococcus mitis*, an oropharyngeal microbe known to cause pneumonia in HCT recipients and *Corynebacterium propinquum*, one virulent *Corynebacterium* species associated with LRTI (Langelier et al., 2018).

mNGS has also been studied to evaluate its role in adjustment of treatment of immunocompromised patients. mNGS tests using plasma or LRT samples had clinical impacts via new diagnoses, early diagnoses, and initiation of therapy for fungal infection; de-escalation of therapies; and by ruling out infectious etiologies (Hogan et al., 2021; Sun et al., 2021; Zhan et al., 2021). Moreover, mNGS has been implemented to detect viruses in blood plasma samples of immunocompromised patients in recent years (Zanella et al., 2021). Although mNGS is usually used as a tool to detect pathogens in the infectious disease, it can also be used to discover the drug resistance genes by analyzing the microbial nucleic acid sequence messages, which will help clinicians to choose precise antimicrobial agents (Chiu and Miller, 2019; Casto et al., 2021). However, mNGS diagnosis platforms are based on short read sequencing, it is challenging to determine the detected antibiotic resistance genes originated from the genome of the causative pathogen rather than normal flora, or contaminations in environment (Diao et al., 2022). In this study, the antibiotic resistance genes detected by mNGS are for the reference of independent multidisciplinary panel after the initial evaluation on the risk factors of multidrug-resistant bacteria infection.

There are dozens of specialized NGS companies in China, and mNGS has been increasingly implemented in the clinic. Although rapid, sensitive and specific microbiology diagnostic method-based antimicrobial therapy has been widely used to treat immunocompromised patients, there is still no data on the effects of this newly developed approach on the clinical outcomes of immunocompromised patients with SCAP. Therefore, we designed this prospective, multicenter, randomized controlled trial of mNGS-guided antimicrobial therapy compared with conventional antimicrobial therapy in this population. As far as we know, this is the first clinical trial to focus on effects of mNGS-based antimicrobial therapy on the clinical prognosis in an immunocompromised population. One limitation of our study is that the study is an open-label trial, but we think this will not affect our primary outcome measurements.

## Conclusion

The MATESHIP study will provide high quality evidence to show the effects of mNGS in guiding treatment for immunocompromised patients with SCAP. We believe that mNGS-guided antimicrobial treatment for immunocompromised patients with SCAP will improve clinical outcomes among these patients.

## Trial status

The trial is recruiting study subjects.

## Trial sponsor

The trial sponsor is Qilu hospital of Shandong University, Jinan, China.

## Data availability statement

The original contributions presented in this study are included in the article/Supplementary material, further inquiries can be directed to the corresponding author.

## Ethics statement

The studies involving human participants were reviewed and approved by Medical Ethics Committees of Qilu Hospital of Shandong University. The patients/participants provided their written informed consent to participate in this study.

## Author contributions

SF and HW wrote the protocol and initiated the study. HW was sponsor and managed the trial. NX contributed to the statistical analysis. HW supervised the manuscript. All authors collected the data, read and approved the final manuscript.

## Footnotes

1
http://www.R-project.org/
