# Supplementary material for: Metagenomic next-generation sequencing-guided antimicrobial treatment versus conventional antimicrobial treatment in early severe community-acquired pneumonia among immunocompromised patients (MATESHIP): A study protocol
Source: Front Microbiol. 2022 Aug 2;13:927842. doi: 10.3389/fmicb.2022.927842 (PMC9379097; doi:10.3389/fmicb.2022.927842)
Supplement: Supplementary file 1 [file Data_Sheet_1.docx]

Supplementary Materials

**Metagenomic Next-generation Sequencing**-**guided Antimicrobial Treatment Versus Conventional Antimicrobial Treatment in Early Severe Community-acquired Pneumonia Among Immunocompromised Patients: A Study Protocol**

The supplementary material includes Initial Empirical Treatment in Immunocompromised Patients,

Metagenomic NGS Measurement Details and 2 supplementary Figures.

- Initial Empirical Treatment in Immunocompromised Patients
- Metagenomic NGS Measurement Details
- Figure 1
- Figure 2

**Initial Empirical Treatment in Immunocompromised Patients**

As immunocompromised patients have different types of pre-existing immune dysfunction and unique immunological risk, they often need individualized empirical treatments. According to a consensus statement regarding initial strategies for immunocompromised patients with CAP (Ramirez et al., 2020) and 2021 International Guidelines for Management of Sepsis and Septic Shock (Evans et al., 2021), the participants will be treated individualized depending on the clinical status, medical history, laboratory results and imaging patterns. Briefly, the core respiratory pathogens, including gram-positive bacteria (*Streptococcus pneumoniae, Staphylococcus aureus* (MSSA)*, Streptococcus pyogenes,* other *Streptococci*), gram-negative bacteria (*Haemophilus influenzae, Moraxella catarrhalis, Enterobacteriaceae* (eg, *Klebsiella species, Escherichia coli*), “atypical” bacteria (*Legionella pneumophila, Chlamydophila pneumoniae, Mycoplasma pneumoniae, Coxiella burnetii*), and respiratory viruses (Influenza virus, Parainfluenza virus, et al), are taken into account for the empirical antimicrobial treatment by the attending physician. Additional empirical treatment should be considered according to the presence of risk factors for drug-resistant or opportunistic pathogens. According to their recent history of colonization/infection and the other risk factors of methicillin-resistant *Staphylococcus aureus* (MRSA) or multiple drug resistance (MDR) gram-negative bacilli (Evans et al., 2021), we will choose initial empiric therapy to cover the above pathogen, followed by rapid de-escalation if no multidrug-resistant pathogen is identified. Initial therapy will cover *pneumocystis jirovecii* for patients with one of the following conditions: 1. HIV host; 2. CD4 counts less than 200 cells/μl (or percentage lower than 14%); 3. severely impaired cell-mediated immunity; 4. imaging patterns with diffuse, bilateral, interstitial infiltrates or alveolar opacities. For patients with prolonged neutropenia and have radiographic features of filamentous fungus infection, we will be careful of filamentous fungi such as *Aspergillus*, *Mucorales*. Moreover, for patients with heart, lung, liver, or hematopoietic stem cell transplant and without prophylaxis with TMP-SMX, when they have CAP and evidence for a lung or brain abscess, we will consider the possibility of *Nocardia* infection. About virus, when patients have a vesicular rash, we will cover varicella-zoster virus; and when patients have a recent lung transplant or hematopoietic stem cell transplant, or suspected *pneumocystis jirovecii* infection, we will cover cytomegalovirus. When we get the results of microbiologist tests, we will alter or confirm a definitive treatment.

Reference

Evans, L., Rhodes, A., Alhazzani, W., Antonelli, M., Coopersmith, C. M., French, C. et al. (2021) Surviving sepsis campaign: international guidelines for management of sepsis and septic shock 2021. Intensive Care Med. 47, 1181-1247.

Ramirez, J. A. and Musher, D. M. et al. (2020). Treatment of Community-Acquired Pneumonia in Immunocompromised Adults: A Consensus Statement Regarding Initial Strategies. *Chest* **158** (5): 1896-1911.

**Metagenomic NGS Measurement Details**

**Metagenomic NGS and Analysis**

***Nucleic Acid Extraction, Library Preparation, and Sequencing***

Total DNA and RNA will be respectively extracted from LRT samples using QIAamp UCP Pathogen DNA Kit (Qiagen) and QIAamp Viral RNA Kit (Qiagen). Human DNA will be removed using Benzonase (Qiagen) and Tween20 (Sigma), and ribosomal RNA (rRNA) will be removed using Ribo-Zero rRNA Removal Kit (Illumina) before library preparation. Then cDNA will be generated using reverse transcriptase and dNTPs (Thermo Fisher). Libraries will be constructed for the DNA and cDNA samples using a Nextera XT DNA Library Prep Kit (Illumina, San Diego, CA). The quality of the libraries will be assessed by an Agilent 2100 Bioanalyzer. Qualitied libraries then will be sequenced using Illumina Nextseq 550 sequencer platform (75-bp single-end reads, Illumina).

***Bioinformatic Analysis***

High-quality sequencing data will be generated by removing low-quality, adapter contamination, duplicate reads, and shorter than 50 bp reads using Trimmomatic, followed by computational subtraction of human host sequences mapped to the human reference genome (hg38) using Burrows-Wheeler Aligner software (Li and Durbin, 2009). A set of criteria have been designed for selecting representative assembly for microorganisms (bacteria, viruses, fungi, protozoa, and other multicellular eukaryotic pathogens) from the NCBI Nucleotide and Genome databases (<ftp://ftp.ncbi.nlm.nih.gov/genomes/>). The final database consists of about 13000 genomes. Microbial reads will be aligned to database with SNAP v1.0beta.18(Zaharia et al., 2011). Virus-positive detection results (DNA or RNA viruses) is defined as the coverage of three or more non-overlapping regions on the genome. Bacteria, fungi and parasites will be reported positive detection for a given species or genus if the reads per million (RPM) ratio or RPM-r (the RPM corresponding to a given species or genus in the clinical sample divided by the RPM in the NC/negative control) is ≥5 (Miller and Naccache et al., 2019). For negative controls, Peripheral blood mononuclear cell samples with 10^5^ cells/mL from healthy donors will be prepared in parallel with each batch, using the same protocol, and sterile deionized water will be extracted alongside the specimens to serve as non-template controls (Miller and Naccache et al., 2019).

In addition, antibiotic resistant genes (ARGs) will also be identified. We will use a big data to find out correlation between pathogen reads and ARGs reads, then we build up a model to predict which pathogen this ARG belongs to. The software DeepARG (Arango-Argoty and Garner et al., 2018) will be used to detect ARGs.

Reference

Li, H. and Durbin, R. (2009). Fast and accurate short read alignment with Burrows-Wheeler transform. *Bioinformatics*. 25 (14): 1754-60.

Miller, S. and Naccache, S. N. et al. (2019). Laboratory validation of a clinical metagenomic sequencing assay for pathogen detection in cerebrospinal fluid. *Genome Research*. 29 (5): 831-842.

Salter, S. J. and Cox, M. J. et al. (2014). Reagent and laboratory contamination can critically impact sequence-based microbiome analyses. *BMC Biol*. 12: 87.

Zaharia, M., Bolosky, W.J., Curtis, K., Fox, A., Patterson, D., Shenker, S., et al. Cornell University. (2011). Faster and more accurate sequence alignment with SNAP. <https://arxiv.org/abs/1111.5572.> [Accessed November 23, 2011].

Chiu, C. Y. and Miller, S. A. (2019). Clinical metagenomics. *Nat Rev Genet*. 20 (6): 341-355.

Gu, W., Miller, S., Chiu, C.Y. (2019). Clinical Metagenomic Next-Generation Sequencing for Pathogen Detection. *Annu Rev Pathol*. 14, 319-388.

Arango-Argoty, G. and Garner, E. et al. (2018). DeepARG: a deep learning approach for predicting antibiotic resistance genes from metagenomic data. *Microbiome*. 6 (1): 23.

Liu, H. and Zhang, Y. et al. (2022). Application of mNGS in the Etiological Analysis of Lower Respiratory Tract Infections and the Prediction of Drug Resistance. *Microbiol Spectr*. 10 (1): e0250221.

## Supplementary Figures


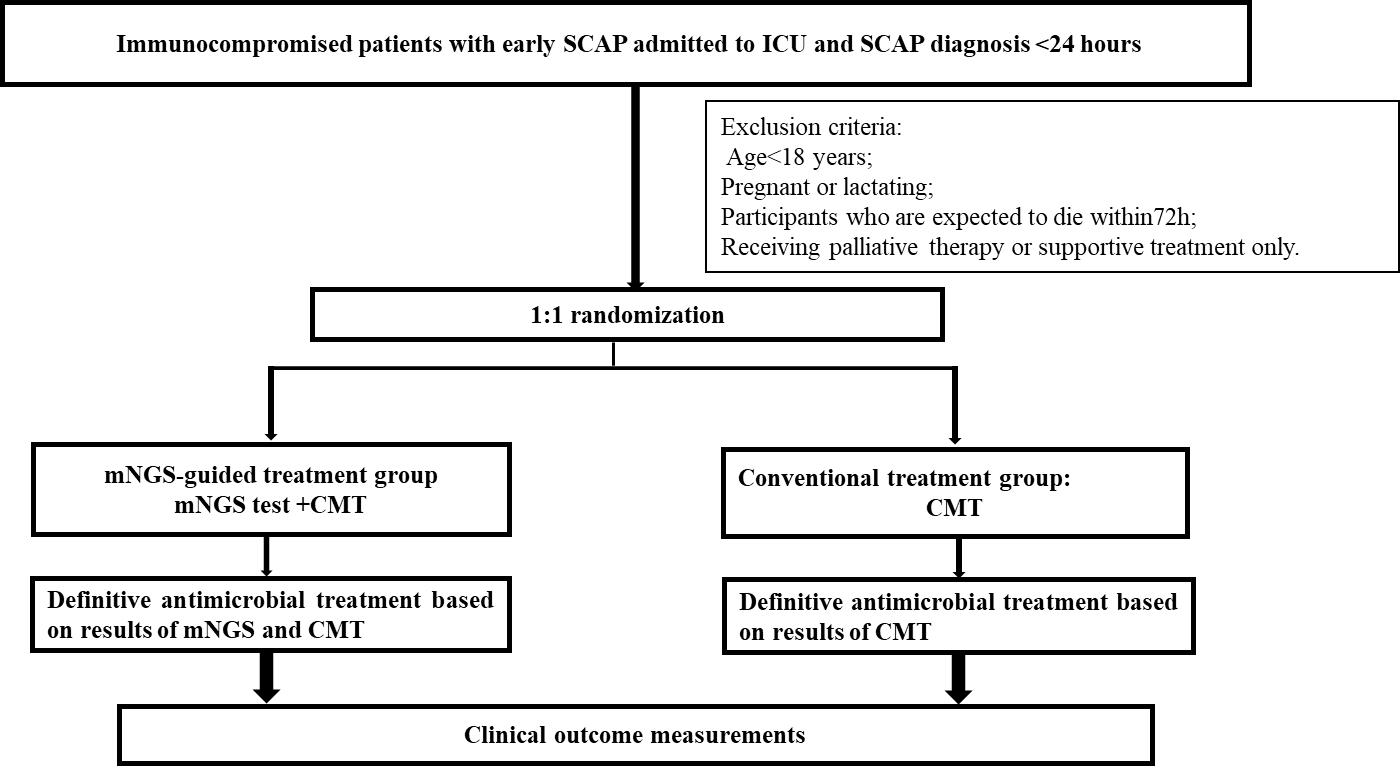


**Supplementary Figure 1.** Flowchart of the study protocol. SCAP: severe community acquired pneumonia, ICU: intensive care unit, mNGS:

metagenomic next-generation sequencing, CMT: conventional microbiological tests.


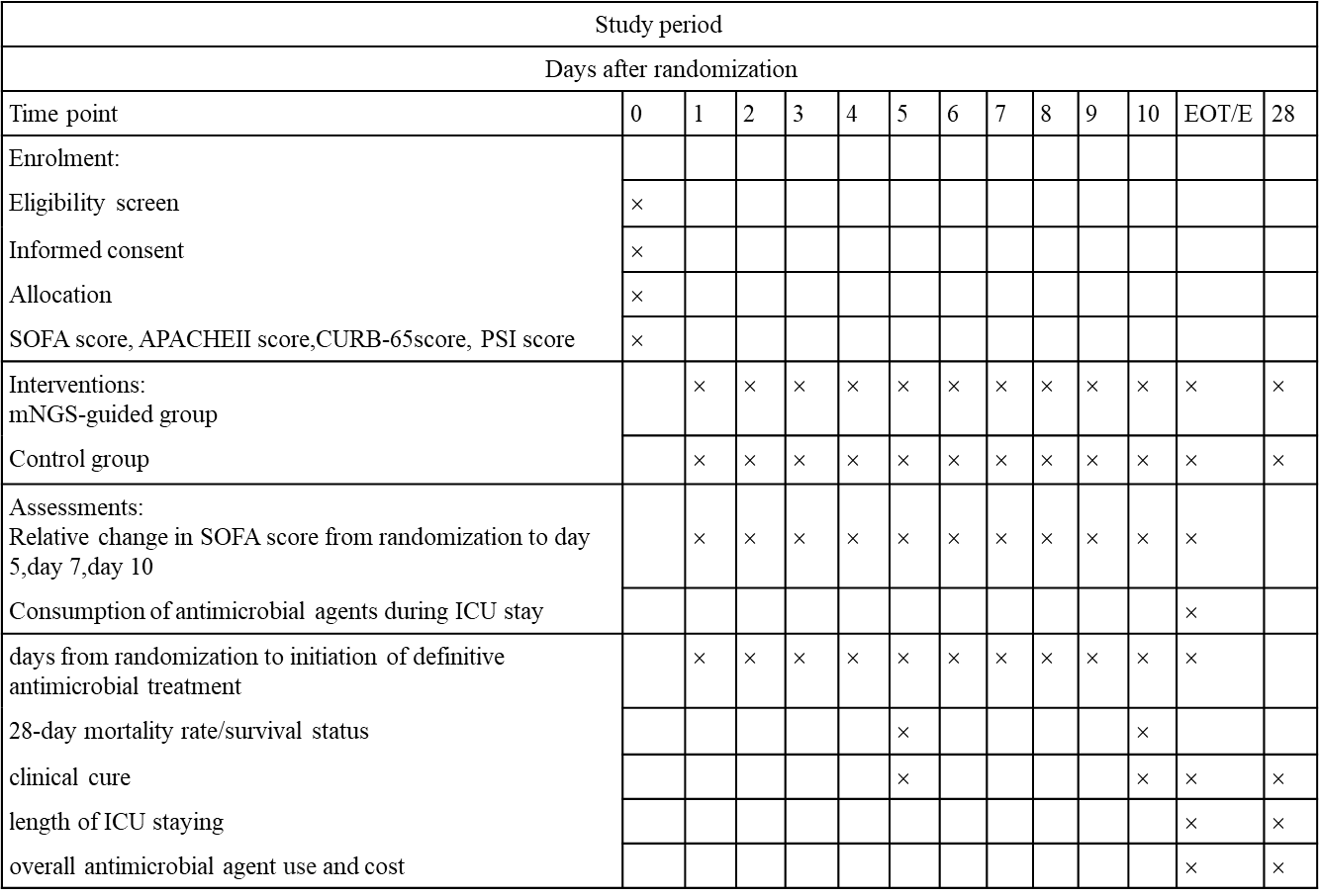


**Supplementary Figure 2.** Schedule of enrolment, interventions, and assessments. EOT: End of therapy; E: Discharge from the ICU.
